# Segment-specific association of carotid-intima-media thickness with cardiovascular risk factors – findings from the STAAB cohort study

**DOI:** 10.1186/s12872-019-1044-0

**Published:** 2019-04-04

**Authors:** Lara Müller-Scholden, Jan Kirchhof, Caroline Morbach, Margret Breunig, Rudy Meijer, Viktoria Rücker, Theresa Tiffe, Tino Yurdadogan, Martin Wagner, Götz Gelbrich, Michiel L. Bots, Stefan Störk, Peter U. Heuschmann

**Affiliations:** 1 Comprehensive Heart Failure Center Würzburg, University and University Hospital Würzburg, Straubmühlweg 15, 97080 Würzburg, Germany; 2 Department of Internal Medicine I, University Hospital Würzburg, Würzburg, Germany; 3 Julius Center for Health Sciences and Primary Care, University Medical Center Utrecht, Utrecht, The Netherlands; 4 Institute of Clinical Epidemiology and Biometry, University of Würzburg, Würzburg, Germany; 5 Clinical Trial Center, University Hospital Würzburg, Würzburg, Germany

**Keywords:** Carotid intima-media thickness (CIMT), Cardiovascular risk prediction, Carotid segment, Carotid ultrasound, Cardiovascular risk factors

## Abstract

**Background:**

The guideline recommendation to not measure carotid intima-media thickness (CIMT) for cardiovascular risk prediction is based on the assessment of just one single carotid segment. We evaluated whether there is a segment-specific association between different measurement locations of CIMT and cardiovascular risk factors.

**Methods:**

Subjects from the population-based STAAB cohort study comprising subjects aged 30 to 79 years of the general population from Würzburg, Germany, were investigated. CIMT was measured on the far wall of both sides in three different predefined locations: common carotid artery (CCA), bulb, and internal carotid artery (ICA). Diabetes, dyslipidemia, hypertension, smoking, and obesity were considered as risk factors. In multivariable logistic regression analysis, odds ratios of risk factors per location were estimated for the endpoint of individual age- and sex-adjusted 75th percentile of CIMT.

**Results:**

2492 subjects were included in the analysis. Segment-specific CIMT was highest in the bulb, followed by CCA, and lowest in the ICA. Dyslipidemia, hypertension, and smoking were associated with CIMT, but not diabetes and obesity. We observed no relevant segment-specific association between the three different locations and risk factors, except for a possible interaction between smoking and ICA.

**Conclusions:**

As no segment-specific association between cardiovascular risk factors and CIMT became evident, one simple measurement of one location may suffice to assess the cardiovascular risk of an individual.

## Background

Many cardiovascular (CV) events occur in thitherto asymptomatic patients [1]. Therefore, it is important to improve assessment of subclinical vascular disease – especially in subjects with an intermediate risk according to established risk prediction models [2, 3]. Measuring carotid intima-media thickness (CIMT) via B-mode ultrasound presents a widely accepted, noninvasive, sensitive and reproducible technique to quantify subclinical vascular disease [2, 4, 5]. It is accepted that CIMT at three different locations, i.e. common carotid artery (CCA), carotid bulb, and internal carotid artery (ICA), is associated with established CV risk factors [6]. CIMT is also a generally acknowledged independent predictor for the occurrence of CV disease [7, 8]. Furthermore, interventional studies have shown that CIMT progression can be positively affected by appropriate treatment of CV risk factors [9]. However, current guidelines do not recommend the use of CIMT for systematic risk assessment in clinical practice for primary prevention [10, 11]. This advice was based on meta-analyses failing to detect a clinically relevant improvement in the performance of common prediction models by adding CIMT [12, 13]. Of note, these meta-analyses solely relied on CIMT of the CCA, and measurements of the bulb and/or ICA were not taken into consideration [14]. Further, these guidelines are controversially discussed as plaque prevalence, known as another important risk modifier, differs among sites [14]. Finally, the association between CIMT and risk factors as systolic blood pressure, cholesterol levels or smoking, seems to show a segment-specific effect [6, 15, 16]. Most of the other studies analyzing the segment-specific effect of different CV risk factors on CIMT originated from US populations [6, 15, 16]. These results are not readily generalizable to European populations because of different ethnicities and lifestyles.

Thus, the aim of this study was to examine whether there is a segment-specific association between the traditional CV risk factors and CIMT measured at different locations of the carotid artery in a representative sample from a German population. Furthermore, we aimed to assess, which segment might best reflect the impact of CV risk factors.

## Methods

### Subjects

The study sample derived from the population-based STAAB (Characteristics and Course of Heart Failure Stages A-B and Determinants of Progression) cohort study. The study design has been reported in detail previously [17]. Briefly, STAAB includes 5000 people to assess the prevalence of heart failure stages A and B, and to investigate the progression from asymptomatic cardiac dysfunction into symptomatic heart failure [17]. Subjects were drawn randomly from a sample of the general population of the city of Würzburg. Subjects had to be aged between 30 and 79 years at the day of sampling. The only exclusion criterion was a pre-existing diagnosis of symptomatic heart failure. Baseline assessment took place from December 2013 to October 2017. All study procedures follow a priori defined measurement protocols are subjected to rigid quality control.

### Risk factor assessment

Five risk factors were considered and the following definition were used. Manifest diabetes mellitus was assumed if the level of the HbA1c was above 6.5%, or fasting plasma glucose level was above 7 mmol/l, or if the subject was on anti-diabetic medication. Dyslipidemia was defined as total blood cholesterol level above 200 mg/dl after a fasting period of at least 10 h, or use of lipid lowering drugs. Arterial hypertension was defined as blood pressure above 140/90 mmHg or use of anti-hypertensive medication. All participants, who were current smokers or ex-smokers (defined as having smoked at least 100 cigarettes in their lifetime) at the time of examination, were counted as smokers. Smoking of cigars or pipe was also considered. Subjects were categorized as obese with a body mass index (BMI) above 30 kg/m^2^. Blood pressure was the median of two to three measurements taken five minute apart in sitting position. Definitions of risk factors were used in previous publications from this study population [17, 18]. A subcohort of apparently healthy people free of any CV risk factor and without previous stroke or CV disease was also defined, and age- and sex-adjusted reference values of CIMT were generated for the population. In accordance with current guidelines, the 75th percentile was considered as cut-off-point indicating an increased individual risk for the manifestation of CV disesease [2].

### Carotid ultrasound

All examiners performing sonographic measurements of subjects underwent an extended training and certification process conducted by an independent expert (RM) prior to the first examination. The training protocol included acquisition, storage, and analysis of the data following published standards [2]. Two different ultrasound devices were used to image the arteries (Vivid S and Vivid Q, General Electric Healthcare). CIMT was assessed via B-mode sonography using a 10 (Vivid Q) or 13 MHz (Vivid S6) linear transducer (8 L-RS). Before the exact measurement of CIMT, a standardized screening of the carotid artery was performed, and the existence and distribution of plaques were captured. The far wall of three different, well-defined locations at both sides of the neck was measured during the end of diastole. The CCA was defined as the segment 10 mm prior to the beginning of the bifurcation. The bulb was defined as the segment from the beginning to the tip of the flow divider and ICA as the segment 10 mm after the tip of the flow divider. Pulsed wave Doppler and color Doppler were used to distinguish the internal from the external carotid artery. Each segment image was captured at the highest visible IMT using the ECG R-wave (end diastole). Plaques were defined as CIMT > 1.5 mm in one or more of the measured CIMT segments, and were included in the measurement. Images were stored and analyzed off-line using the Syngo Arterial Health Package (Syngo US Workplace, Siemens Medical Solutions USA, Inc.). A composite CIMT value was calculated as the mean of all available values (one to six). Reference values were generated for every location and the composite value. The average intraclass correlation coefficient (ICC) for interobserver reproducibility based on 40 double measurements was 0.78 (95% CI: 0.62–0.87) for composite CIMT, 0.67 (95% CI: 0.48–0.82) for right CCA, 0.63 (95% CI: 0.36–0.80) for right bulb, 0.65 (95% CI: 0.42–0.80) for right ICA, 0.65 (95% CI: 0.42–0.80) for left CCA, 0.63 (95% CI: 0.37–0.80) for left bulb, and 0.31 (95% CI: 0.01–0.57) for left ICA, respectively.

### Statistical analysis

For the comparison of continuous variables, t-test for independent samples after checking Levene’s test for equality of variances was used. The chi-square test was used for the comparison of categorical variables. Multivariable logistic regression analysis was conducted using an inclusion-model considering diabetes, dyslipidemia, hypertension, smoking and obesity all in the same model. As outcome variable, the age- and sex-adjusted normative value above the 75th percentile was used for each location. A sensitivity analysis excluding apparently healthy subjects did not result in material changes of the estimates. Thus, the data of all subjects were included in the final analysis. Collinearity of CV risk factors was tested by computing the variance inflation factors (cut off value: 2). The age spectrum was divided into three equal age groups. Interactions between the different locations, the different age groups and the different risk factors were analyzed using generalized estimating equations (GEE). Logarithms of IMT values were computed to ensure standard distribution and used as dependent factor. For each risk factor a distinct age- and sex-adjusted model was calculated. All statistical analyses were performed using SPSS (version 24). *P*-values < 0.05 were considered statistically significant.

## Results

Between December 12, 2013 and March 1, 2016, the first 2492 people were examined. This number corresponds to the first 19 batches of the recruitment process. No CIMT values were available for 7% of the study sample. Distribution of missing values in the respective distinct location did not show a statistically significant association with the different locations or sides (right side: CCA 9%, bulb 10%, ICA 10%; left side: CCA 9%, bulb 9%, ICA 10%; all *p* > 0.05). The general characteristics of the population are shown in Table 1. A total of 76% of the whole population had at least one risk factor.

**Table 1:** Characteristics of the study population

Variable	Total *n* = 2492
Men, *n* (%)	1212 (49)
Age [years], mean (SD)	54 ± 12
Agegroups *n* (%)	
< = 45 years	597 (24)
46–60 years	1021 (41)
> 60 years	868 (35)
Height [cm], mean (SD)	171 ± 9
Weight [kg], mean (SD)	78 ± 17
Obesity [BMI > 30 kg/m], *n* (%)	492 (20)
Blood pressure systolic [mmHg], mean (SD)	131 ± 18
Blood pressure diastolic [mmHg], mean (SD)	79 ± 10
Hypertension, *n* (%)	879 (38)
Total cholesterol [mg/dl], mean (SD)	208 ± 38
Dyslipidemia, *n* (%)	338 (15)
HbA1c [%], mean (SD)	5.5 ± 0.61
Diabetes, *n* (%)	217 (9)
Current or ex-smoker, *n* (%)	1324 (54)
Current smoker, *n* (%)	455 (19)
Ex-smoker, *n* (%)	869 (35)
Previous cardiovascular disease, *n* (%)	102 (4)
Previous stroke, *n* (%)	49 (2)
Apparently healthy, *n* (%)	538 (24)

The composite CIMT was 0.68 ± 0.17 mm for the overall population. Men had higher CIMT values than women in every location. CIMT was highest at the bulb, followed by CCA, and lowest at the ICA. As there were no differences between the IMT values of both sides of the neck (*p *> 0.05), mean values of both sides were calculated and used as independent variables for the following analysis. People with previous stroke, CV event or manifest peripheral arterial disease had higher CIMT values in any considered location (all *p* < 0.001). Plaque prevalence was relatively low with a total number of 586 people (26%) having at least one plaque in the investigated carotid artery segment (Table 2).

**Table 2:** Segment-specific assessment of CIMT

Variable	Total n = 2492
Mean RCCA [mm], mean (SD)	0.66 ± 0.17
Men [mm], mean (SD)	0.68 ± 0.18
Women [mm], mean (SD)	0.64 ± 0.15
Mean right bulb [mm], mean (SD)	0.82 ± 0.34
Men [mm], mean (SD)	0.88 ± 0.37
Women [mm], mean (SD)	0.78 ± 0.31
Mean RICA [mm], mean (SD)	0.58 ± 0.22
Men [mm], mean (SD)	0.61 ± 0.24
Women [mm], mean (SD)	0.55 ± 0.20
Mean left CCA [mm], mean (SD)	0.66 ± 0.18
Men [mm], mean (SD)	0.68 ± 0.18
Women [mm], mean (SD)	0.63 ± 0.16
Mean left bulb [mm], mean (SD)	0.82 ± 0.32
Men [mm], mean (SD)	0.87 ± 0.35
Women [mm], mean (SD)	0.76 ± 0.27
Mean left ICA [mm], mean (SD)	0.57 ± 0.21
Men, [mm], mean (SD)	0.61 ± 0.23
Women, [mm], mean (SD)	0.54 ± 0.17
Mean of both sides	
CCA [mm], mean (SD)	0.66 ± 0.15
Bulb [mm], mean (SD)	0.82 ± 0.28
ICA [mm], mean (SD)	0.57 ± 0.18
Composite CIMT [mm], mean (SD)	0.68 ± 0.17
Plaque – *n* (%)	586 (26)
CCA	40 (2)
Bulb	521 (23)
ICA	137 (6)

Multivariable logistic regression analysis showed that arterial hypertension, dyslipidemia and smoking were independent predictors for CIMT values above the age- and sex-adjusted 75th percentile (Table 3). While hypertension showed a significant effect on all locations measured, dyslipidemia demonstrated a statistically significant effect only at the bulb and ICA. In addition, a strong positive trend for the association of dyslipidemia with CCA was found. The effects of smoking were visible at the bulb and CCA. Diabetes mellitus and obesity seemed to exert no effects on any segments. Odds ratios for significant risk factors varied between 1.30 (for smoking and CCA) and 1.86 (for hypertension and composite CIMT). No collinearity between the different CV risk factors was apparent. In GEE we found a significant interaction between the segment of measurement and the following risk factors: dyslipidemia (*p* < 0.001), hypertension (*p* < 0.001), and smoking (*p* = 0.002). No interaction was found between age in tertiles and the five major risk factors (all *p* > 0.05).

**Table 3:** Strength of the association between CV risk factor and CIMT value above the 75th percentile

Risk factor	Composite CIMT	CCA	Bulb	ICA
Diabetes	1.24 (0.89–1.71)	1.22 (0.87–1.70)	1.10 (0.78–1.54)	1.29 (0.91–1.80)
Dyslipidemia	1.73 (1.34–2.23)	1.28 (0.98–1.65)	1.79 (1.38–2.32)	1.43 (1.10–1.86)
Hypertension	1.86 (1.53–2.27)	1.66 (1.35–2.03)	1.75 (1.44–2.13)	1.45 (1.22–1.84)
Obesity	0.91 (0.72–1.16)	0.95 (0.74–1.21)	0.88 (0.69–1.12)	1.25 (0.98–1.60)
Smoking	1.47 (1.23–1.77)	1.30 (1.08–1.57)	1.44 (1.20–1.72)	1.01 (0.84–1.22)

Data are odds ratio (OR) with 95% confidence interval (CI) derived from multivariable regression analysis for the likelihood of having CIMT values above the 75th percentile

## Discussion

We analyzed the association between CV risk factors and different locations of CIMT in a population-based study from the general population of Würzburg, Germany. Our study comprised not only a broad age range but also a detailed assessment of CV risk profile and CIMT. Hypertension and dyslipidemia showed a significant association on having CIMT above the 75th percentile independent from the measurement location, while smoking seemed to have an effect on CCA and bulb only. Our results imply that no segment carries more information about the influence of CV risk factors on CIMT than the others.

In the STAAB cohort the prevalence of plaque was relatively low, with 26% of all people having at least one plaque in their carotid arteries. Other studies published plaque prevalences between 34 and 87% [19–21]. This prompts the question as to whether the plaque prevalence and lower CIMT is attributable to the reported population or if the methods of plaque acquisition differ [20, 22]. One possible explanation for a lower prevalence of plaques observed here might be variations in uptake of preventive medication such as lipid-lowering drugs [18]. CIMT values were higher in men than women in all different locations with the highest CIMT values found in the bulb [6, 23]. Other population studies reported the lowest values in the CCA, whereas in our study the lowest values were found in the ICA [23, 24]. As plaque prevalence in ICA is usually higher than in the CCA and plaque prevalence in the STAAB cohort is comparatively low this could explane this difference [25]. In line with previous studies, there was no difference between CIMT of right and left carotid arteries [26].

ICC for left internal carotid artery was remarkably low compared to the other segments (0.31 vs. 0.63 (right bulb) - 0.78 (composite CIMT)). Nichols et al. [27] report the best matches for the lateral images of the right side, therefore the handiness of the examiner could play a role since the right IMT is examined with the right hand and the left with the left and all examiners were right-handed. Regarding the particularly low ICC of the left internal carotid artery, this might be attributable to the fact that this segment is the most challenging for a right handed examiner.

Taking all CV risk factors in consideration, only diabetes and obesity seemed to have no association with CIMT. With regard to obesity, many studies also found no independent relation with CIMT as well [6, 28]. Apparently, the known higher CV risk of obese people cannot be simply read off the CIMT [10]. BMI is believed to have no direct influence on CIMT, but rather may effect CIMT through other risk factors [28]. Further, we were unable to show a statistically significant effect of diabetes on CIMT. The pathophysiological concept renders glycated molecules in the arteries responsible for the observed greater CV risk of diabetic people, but in epidemiological studies this association is infrequently present [29, 30]. The reasons for this finding are unclear and might be caused by the low number of cases or by collinearity of different factors that in multivariable analysis masking true effects [29, 30]. In the present study 217 people (9%) did present overt manifest diabetes mellitus. Thus, we consider the number of cases in the different locations as sufficient to detect an effect. In our population, diabetes did not contribute to CIMT thickness in a clinically relevant way, even if nearly 30% of people with an HbA1c > 6.5% were diagnostically naïve and 44% of people with antidiabetic medication in the STAAB population were insufficiently treated [18].

Our findings confirm the importance of the three other risk factors dyslipidemia, hypertension and smoking that showed a significant effect on CIMT [6, 22, 30, 31]. Results of the logistic regression indicate that there was no segment-specific effect of hypertension and dyslipidemia. Only smoking showed a potential interaction with the ICA segment. However, previous studies reported a segment-specific effect and only disagreed on the segment which carries the most information [6, 15, 16]. Polak et al. considered the CCA as the location containing the most information, while Urbina et al. identified the bulb as the most informative segment [15, 16]. There are only a few publications that also did not show a segment-specific influence of the traditional risk factors [26, 32, 33]. Variations between prvious studies might be caused by differences in analytical methodology. In contrast to previous publications we used different statistical methods. Rather than employing linear regression and comparing the magnitude of R^2^ [2], we used logistic regression with the endpoint of CIMT >75th percentile of age- and sex-adjusted reference values [6, 15]. The most important advantage of this approach is that it portrays the clinically relevant effect of a CV risk factor. However, these different approaches render a direct comparison of our results with previous studies problematic. Using a linear analysis to detect possible interactions we did also find significant interactions between the location of measurement and the risk factors dyslipidemia, hypertension and smoking. Nevertheless, Lorenz et al. reported very similar hazard rate ratios for the three different locations and the occurrence of a CV event and concluded, as well as del Sol et al., that there is no difference in the power of risk prediction between segments [26, 33].

## Limitations and strengths

We observed a lower prevalence of plaques than reported by others. Reproducibility of image acquisition and reading is an important element built into the STAAB study design [17, 34]. All technicians underwent a dedicated training and certification procedure and followed a structured protocol. Further, when analyzing the reproducibility at different locations we did not find a segment-specific difference. Nevertheless, as five sonographers were capturing images and reading CIMT along the study, a certain degree of variation must be considered as a potential confounding factor. The subgroup of apparently healthy people, forming the basis for the reference values, compared favourably with similar studies that aimed to generate reference values. The 75th percentile of “normal” values was lower in our sample than in previous studies [20]. This may be partially due to the strict definition of the risk factor “smoker” that included all people ever smoking more than 100 cigarettes or cigars in their life – regardless of the current smoking status. This was based on previous studies reporting an increase in CIMT and progression rates up to 2 or 3 years after smoking cessation [35, 36]. However, this approach excludes people with a very low tobacco consumption, who may not have subclinical atherosclerosis caused by smoking. The size of our study sample provides for sufficient statistical power analyzing subgroups and comparisons with previous population-based studies [15, 29]. However, the generalizability may be higher in groups of middle-aged people with very frequent CV risk factors like hypertension and smoking in contrast to other population groups. As we observed no significant interaction between age groups and risk factor level, we consider it unlikely that the variation in age may be a major confounder of our results.

## Conclusion

The results of our study support the hypothesis that a CIMT measurement derived at a single segment provides sufficient information about the association between risk factors and CIMT. As there is no preference for a specific segment nor side for risk factors, a simple measurement executed at one location on either side of the neck might be sufficient to allow CV risk assessment in an individual. Further research should focus on the value of the segment-specific effects of plaques in risk prediction.

## Abbreviations

CCA: Common carotid artery
CIMT: Carotid intima-media-thickness
CV: Cardiovascular
GEE: Generalized estimating equations
ICA: Internal carotid artery
ICC: Intraclass correlation coefficient
STAAB: Characteristics and course of heart failure stages A-B and determinants of progression
